# Editorial: Advancing vascularized tissue models through biomaterials and biofabrication

**DOI:** 10.3389/fbioe.2024.1518452

**Published:** 2024-11-15

**Authors:** Martin Ehrbar, Silvia Lopa, Chiara Arrigoni

**Affiliations:** ^1^ Clinic for Obstetrics, University of Zürich, Zürich, Switzerland; ^2^ Cell and Tissue Engineering Laboratory, IRCCS Istituto Ortopedico Galeazzi, Milan, Italy; ^3^ Regenerative Medicine Tehcnologies Lab, Ente Ospedaliero Cantonale, Bellinzona, Switzerland; ^4^ Euler Institute, Università della Svizzera Italiana, Lugano, Switzerland

**Keywords:** vascularization, tissue engineering, biofabrication, in vitro models, biomaterials

Vascular networks are integral elements of our body tissues with few exceptions. They undoubtedly play key roles in tissue development, in assuring the transport of substances to and from the tissue, and in tissue regeneration. Therefore, with the aim to advance our understanding of tissue physiology and pathological mechanisms and to regenerate damaged tissues, the generation of functional and vascularized tissue replicates become of high-interest.

However, approaches to reproduce tissue architectures, especially with perfusable vascular systems, remain a significant challenge that is currently addressed with different approaches, ranging from the de-cellularization of tissues and using the pre-existing vascular conduits for reseeding of cells, to the engineering of cell-instructive biomaterials for the embedment of tissue-specific cells, and the use of bio-fabrication techniques allowing three-dimensional arrangement of cells and specific bioinks. While these approaches come with their own advantages and limitations, impressive advances have been made in all of them. For instance, starting from the seminal work on the decellularization and recellularization of vascularized lungs (Ott et al., 2010), several techniques have been developed to achieve the formation of fully re-endothelialized blood vessels in decellularized matrices with pre-existing vascular networks (Wu et al., 2022). Regarding the development of biofabrication techniques for vascularization, even more progresses have been reached, spanning from the development of high resolution bioprinting techniques allowing to reach micrometric dimensions (Brandenberg and Lutolf, 2016) to the fabrication of complex networks exploiting sacrificial materials (Chen et al., 2023), to the combination of small vascularized building blocks in a higher dimension tissue construct (Franca et al., 2023). To further advance the development of vascularized tissue models, likely, the integration of top-down with bottom-up approaches will enable to predefine large vascular channels and to self-assemble vascular capillaries in their vicinity. Additionally, the use of tissue and disease specific cells, including endothelial cells to recapitulate the *in vivo* counterpart, will be required.

In this Research Topic article collection, we present different approaches aimed at achieving tissue models embedding functional vascular networks, applicable to grafts for clinical use and to biological replicates serving as *in vitro* models of different pathologies. In this latter field, cancer models are one of the major applications of vascularized tissue replicates, since many aspects of cancer progression depend on the presence of a functional vascular network. The article from Wang et al. reviews the main approaches that have been leveraged to achieve the generation of a functional vasculature within microfluidic tumor models. Techniques span from self-assembly of endothelial cells to creation of channels through needles and rods to 3D bioprinting approaches. Such vascularized tumor models have been exploited to investigate metastatic progression and tumor angiogenesis in different tumor subtypes, proving as useful tools also for drug discovery.

Beyond the application to *in vitro* models, vascular networks are highly needed also for tissue replicates intended as clinical grafts, since they provide perfusion of oxygen and nutrients to cell embedded in the tissue. One strategy that can be pursued to achieve the formation of these vascular networks is to exploit the vascular system already present in native tissues. To this end, Rougier et al. described an approach for the decellularization of bone grafts which preserved the architecture of the vascular network, leaving a vascular pedicle for anastomosis and for accessing graft vasculature. With their protocol it was possible to efficiently decellularize the tissue, preserving mechanical properties and tissue architecture, including perfusable vessels, and to reseed relevant progenitor cells for the reconstruction of bone tissue.

Although relying on natural vessels assures the presence of a physiological and already formed vascular bed, this strategy is restricted to the use of decellularized tissues, with all the limitations associated to their scarce availability and flexibility. To overcome these limitations, scaffolds based on synthetic or natural biomaterials are a widespread alternative strategy. To achieve the formation of vascular networks inside those grafts it is possible to rely on biofabrication techniques, including the bioprinting of porous bioinks. In this context, Vanlauwe et al. describe the generation of a bioink based on GelMA and blended with low molecular weight alginate as a sacrificial porogen, that can be rinsed from the polymerized scaffold, leaving space for vascularization. The authors demonstrated that even a non-complete dissolution of the porogen increased VEGF availability and, consequently, HUVEC colonization as compared to pristine GelMA.

Finally, once the vascular networks have been generated into engineered grafts, they should persist even after *in vivo* implantation, otherwise leading to dysfunctional grafts. The work from Schwager et al. addressed the problem of vessel regression through the addition of Semaphorin 3A (Sema3A) in the scaffold. Sema3A is known as a key mediator of vascular stabilization, acting through the recruitment of a specific subset of monocytes. Fibrin gels decorated with Sema3A and seeded with stromal vascular fraction cells showed the formation of vascular networks with a longer stability, up to 12 weeks after *in vivo* implantation, as compared to naïve fibrin gels. The improved stability occurred following the recruitment of monocytes positive for neuropilin-1 from mouse blood circulation.

Overall, in this Research Topic we bring different viewpoints on available strategies and still unsolved problems in the realm of vascularization of engineered 3D tissues, a crucial feature that should be achieved for improving their impact and usability.
