# Corrigendum: Editorial: Advancing vascularized tissue models through biomaterials and biofabrication

**DOI:** 10.3389/fbioe.2024.1542997

**Published:** 2025-01-03

**Authors:** Martin Ehrbar, Silvia Lopa, Chiara Arrigoni

**Affiliations:** ^1^ Clinic for Obstetrics, University of Zürich, Zürich, Switzerland; ^2^ Cell and Tissue Engineering Laboratory, IRCCS Istituto Ortopedico Galeazzi, Milan, Italy; ^3^ Regenerative Medicine Tehcnologies Lab, Ente Ospedaliero Cantonale, Bellinzona, Switzerland; ^4^ Euler Institute, Università della Svizzera Italiana, Lugano, Switzerland

**Keywords:** vascularization, tissue engineering, biofabrication, *in vitro* models, biomaterials

In the published article, there was an error in the **Funding** statement, as the funding agency supporting the work of Silvia Lopa was not reported.

“The author(s) declare that no financial support was received for the research, authorship, and/or publication of this article.”

The correct Funding statement appears below.

